# Draft Genome Sequence of *Bacillus* Species from the Rhizosphere of the Desert Plant *Rhazya stricta*

**DOI:** 10.1128/genomeA.00957-15

**Published:** 2015-11-05

**Authors:** S. E. M. Abo-Aba, Jamal S. M. Sabir, Mohammed N. Baeshen, Meshaal J. Sabir, Mohammed H. Z. Mutwakil, Nabih A. Baeshen, Rosalinda D’Amore, Neil Hall

**Affiliations:** a Department of Biological Sciences, Faculty of Science, Biotechnology Research Group, King Abdulaziz University, Jeddah, Saudi Arabia; b Institute for Integrative Biology, University of Liverpool, Liverpool, United Kingdom

## Abstract

In order to better understand the ecology and diversity of microbes in the rhizosphere of desert plants, we undertook a survey of *Bacillus* species isolated from soil around *Rhazya stricta* plants from the area around Jeddah, in The Kingdom, Saudi Arabia. We have sequenced the genomes of 8 *Bacillus* isolates representing four different species.

## GENOME ANNOUNCEMENT

*Bacillus* is the most frequently isolated genus from soil. Several *Bacillus* species are ubiquitous and broadly adapted to grow in diverse settings within the biosphere. *Bacillus* species can be isolated in greater numbers than most other spore forming bacteria from the rhizosphere of a variety of plants, and there is evidence that through these associations they can promote plant growth (1–3). Desert soils are known to have heterogeneous microbial biodiversity heavily influenced by local areas of water availability (4). It has been shown that areas of vegetation have a greater microbial species diversity (5).

As part of a wider study to study the ecology of the rhyzobiome associated with the desert plant *Rhazya stricta* growing near Jeddah in Saudi Arabia, we generated draft genome sequencing of 12 *Bacillus* isolates collected from soil samples. One gram of soil from each collected sample was transferred into a 10 mL tube containing saline solution (NaCl 0.1 w/v) and left for 30 min at room temperature until the soil particles settled. Sample solutions were diluted 10-fold, spread onto LB agar plates, and incubated overnight at 37°C. Bacilli like colonies were isolated according to their morphological characters. Individual colonies from each site were picked up and purified by re-streaking. They were then grown in 10 mL liquid Luria broth (LB), and DNA was extracted using QIAamp minikits (Qiagen).

Genomic DNA was fragmented to approximately 500 bp using Covaris sonicator and TruSeq adaptors (Illumina) ligated to the DNA fragments using the supplied protocol. Each sample was barcoded with sequence-specific adaptors, and the fragments sequenced on a single MiSeq run using forward and reverse 150 bp reads. The sequences were processed to remove low-quality bases using cutadapt (6) and Sickle (https://github.com/najoshi/sickle). Reads were assembled using Velvet (7) using a k-mer length of 71 and annotated using Prokka (8). Whole-genome phylogenies using kSNP were used to assign species (9).

The assembly sizes and number of reads generated are as follows: *Bacillus subtilis* JRS2 (4,060,709 bp, 1,468,268 reads), *Bacillus pumilus* JRS3 (3,758,903 bp, 1,258,270 reads), *Bacillus amyloliquefaciens* JRS5 (4,031,481 bp, 1,526,086 reads), *Bacillus subtilis* JRS6 (3,993,757 bp 1,716,674 reads), *Bacillus subtilis* JRS7 (4,116,767 bp, 1,647,846 reads), *Bacillus amyloliquefaciens* JRS8 (4,090,896 bp, 1,491,538 reads), *Bacillus subtilis* JRS9 (4,044,356 bp, 1,296,858 reads), *Bacillus subtilis* JRS11 (3,962,286 bp 1,678,670 reads).

Our study demonstrates that there is a diverse group of *Bacillus* species present in the rhizosphere of the desert plant *R. stricta*. The genomes were highly conserved with other previously sequenced species with small differences in accessory genes associated with processes such as iron uptake, nutrient transport and nitrate metabolism.

### Nucleotide sequence accession numbers.

The complete genome sequences and annotations are deposited at EMBL, EBI under the study accession no. PRJEB9876 and the following assembly accession numbers: *Bacillus subtilis* JRS2, CYHJ01000001 to CYHJ01000114; *Bacillus pumilus* JRS3, CYHK01000001 to CYHK01000108; *Bacillus amyloliquefaciens* JRS5, CYHL01000001 to CYHL01000128; *Bacillus subtilis* JRS6, CYHN01000001 to CYHN01000080; *Bacillus subtilis* JRS7, CYHO01000001 to CYHO01000211; *Bacillus amyloliquefaciens* JRS8, CYHP01000001 to CYHP01000167; *Bacillus subtilis* JRS9, CYHS01000001 to CYHS01000125; *Bacillus subtilis* JRS11, CYHQ01000001 to CYHQ01000076.
